# Association between personality traits and concerns about falling among older patients: the mediating role of subjective age

**DOI:** 10.3389/fpubh.2024.1343939

**Published:** 2024-08-16

**Authors:** Rongrong Fan, Lili Wang, Wenxiu Wang, Yanan Zhong, Tingting Zhang, Xia Yang, Jing Zhu

**Affiliations:** Department of Pulmonary and Critical Care Medicine, West China Hospital, Sichuan University/West China School of Nursing, Sichuan University, Chengdu, China

**Keywords:** older patients, concerns about falling, personality traits, subjective age, mediating effect

## Abstract

**Background:**

Older patients are at high risk of falling, and regular assessments of their concerns about falling (CaF) are often recommended. The present study aimed to investigate the association between CaF and personality traits among older patients as well as to elucidate the mediating role of subjective age.

**Method:**

A cross-sectional study was conducted among 407 patients aged over 60 years in a tertiary hospital located in Chengdu, Sichuan Province, from March 2023 to May 2023. Predesigned electronic questionnaires were distributed to collect relevant data. Four different models (both crude and adjusted weighted linear regression models) were constructed based on the confounders. Confounders were gradually put into the models to control for bias and to examine the stability of the correlations. Bootstrap sampling was employed to examine the mediating role of subjective age.

**Result:**

According to the fully adjusted model, neuroticism (*β* = 0.17, 95% CI: 0.02 to 0.31, p for trend = 0.02), extraversion (*β* = −0.07, 95% CI: −0.15 to 0.001, p for trend = 0.05), and subjective age (*β* = 2.02, 95% CI: 1.28 to 2.78, p for trend <0.001) were consistently correlated with CaF. Mediating analysis revealed that extraversion was negatively related with CaF both directly and indirectly, via subjective age [23.2% partial effect, bootstrap 95%CI: −0.024(−0.080, −0.000)]. Higher neuroticism was consistently related to older subjective age (*β* = 0.002, 95% CI: 0.001 to 0.004, p for trend = 0.006), while higher levels of conscientiousness, openness, and extraversion were consistently correlated with younger subjective age(*β* = −0.002, p for trend = 0.04; *β* = −0.003, p for trend = 0.003; *β* = −0.002, p for trend = 0.0, respectively).

**Conclusion:**

Extraversion and neuroticism were significantly correlated with CaF. Moreover, subjective age partially mediated the relationship between extraversion and CaF. Furthermore, subjective age was found to be associated with both CaF and personality traits. These findings highlighted the important roles of personality traits and subjective age in assessments of CaF and in the development of strategies for preventing falls among older patients.

## Highlights

- Neuroticism, extraversion, and subjective age were consistently correlated with concerns about falling (CaF).
- Neuroticism directly affected the CaF. Extraversion could directly affect the CaF and could also affect the CaF through mediating effect of subjective age.
- Neuroticism, conscientiousness, openness, and extraversion were significantly correlated with subjective age.

## 1 Introduction

Falls are the second leading cause of unintentional injury-related deaths around the world, and an estimated 684,000 individuals die from falls globally each year (1, 2). Falls lead to substantial increases in morbidity, mortality, healthcare expenses (3), and emergency department admissions (4). Older individuals, especially older patients, face the greatest risk of fatal injury and severe harm resulting from a fall, and these risks increase with advancing age (5). Some guidelines suggest that all older patients should be considered to be high risk for falls (6). Up to one million patients experienced unintentional falls (7), and a significant proportion (80%) of falls occur among patients aged 65 and above (8). Approximately 37.2% of older patients reported at least one fall within the last 12 months, with 66.7% of these patients experiencing fall-related injuries (9).

We contend it is useful to evaluate older individuals’ beliefs, attitudes and priorities regarding falls (6). Concerns about falling (CaF) involve an individual recognizing his or her susceptibility to falls and reflect an emotional response to a perceived threat (10). Adaptive CaF can serve as a catalyst for individuals to make necessary adjustments to their behavior to enhance safety. Maladaptive CaF, such as excessive concern, can result in overly cautious or hypervigilant behaviours that paradoxically increase the risk of injurious falls (11, 12). Conversely, low levels of CaF may indicate an inappropriately high level of confidence in one’s balance. In fact, a significant number of patients are unable to accurately evaluate their susceptibility to falls. A recent review highlighted the inadequate self-perception of fall risk among hospitalized older adults who do not consider themselves at risk for falling (7). This finding was supported by another observational study conducted in China, which revealed that only 61.9% of patients accurately perceived their fall risk, while 27.5% underestimated their risk and 10.6% overestimated it (13). Thus, it may be desirable that clinicians regularly evaluate CaF among older patients (6, 12) as part of multifactorial fall risk assessments. The present study aimed to identify potential factors to help better assess CaF among older patients in clinical practice.

### 1.1 The effect of personality traits on concerns about falling

Personality traits encompass an individual’s pattern of thoughts, feelings, attitudes, habits, and behavior that persist over time and in different situations (14). According to the 5-factor model or Big 5 model, there are five major personality traits: neuroticism, extraversion, openness, agreeableness, and conscientiousness (15). Personality traits are usually stable and constant among older individuals. Differences in personality traits among individuals can lead to variations in emotions (16), expectations (17, 18), motivations, and behaviours (19–21) moreover, differences in personality traits might be related to different beliefs, attitudes and perceptions about falls among older individuals (22, 23). Personality traits may play a role in assessments of CaF. A cross-sectional study conducted in community-dwelling women over 70 years of age revealed that neuroticism was an important psychological factor related to CaF (24). A recent study by Turunen revealed that neuroticism was positively correlated with indoor falls and CaF (25). A prospective investigation of 263 older patients also confirmed that those with higher levels of neuroticism were more prone to experiencing fear of falling within the first 12 weeks following a hip fracture (26). However, the research designs of these studies varied in terms of the participants, settings, and assessment tools. Therefore, evidence supporting the relationship between personality traits and CaF among older patients is limited. Based on the aforementioned findings, we hypothesized that personality traits (especially neuroticism) are significantly related to CaF among older patients (H1).

### 1.2 The mediating role of subjective age

Subjective age refers to an individual’s subjective perception of his or her own age and holds significant relevance as a biopsychosocial indicator within older people and their management. Subjective age reflects a special form of an individual’s self-assessment, self-motivation, self-enhancement, and self-protection in their life (27, 28). Individuals who perceive themselves as younger have reported experiencing better perceived health status, positive coping strategies (29), longer lifespan (30), and a lower risk of dementia (31). On the other hand, older subjective age has been found to be associated with higher levels of depressive symptoms (32), cognitive impairment, and elevated cortisol levels (33). Personality traits have been consistently linked to subjective age in longitudinal studies (34, 35). An open psychological disposition has been found to be associated with feeling younger (36). Specifically, higher levels of extraversion, agreeableness, and conscientiousness have been found to be associated with feeling relatively older over time (37). In contrast, high levels of neuroticism may contribute to an increased likelihood of perceiving oneself as older, partially due to its association with negative health outcomes (38). However, some research has concluded that conscientiousness, neuroticism, and agreeableness are not related to subjective age in older individuals (39). Additionally, the findings of a study by Canada et al. did not support the relationship between extraversion and subjective age (40). The original studies conducted on this topic were limited in scope, and therefore, the evidence remains inconclusive. Herein, we hypothesize that open personality traits (extraversion, openness, agreeableness, and conscientiousness) are linked with younger subjective age (H2). Additionally, previous research has found that subjective age was related to health-related behaviors (36), such as limitations in activities of daily living (ADLs) (41) and CaF. A previous study found a correlation between older subjective age and the development of CaF over time (42). However, in contrast, another study found that CaF was positively linked to a younger subjective age (43). In light of these inconclusive results, we hypothesized that a younger subjective age would be associated with lower CaF (H3).

Previous studies have predominantly concentrated on the direct relationship between personality traits and CaF. The mediating effects of subjective age between these two variables have not been adequately examined. Although the evidence was inconclusive, previous studies have found that subjective age mediated the relationship between personality traits and muscular strength (44) and that muscular strength was closely linked with CaF (45). Therefore, we hypothesize that subjective age mediates the relationship between personality traits and CaF (H4).

### 1.3 The present study

If CaF is an important factor influencing the perceived risk of falling and physical activity among older individuals (46), then it is possible that an accurate assessment of CaF could help to identify older patients with maladaptive perceptions and implement appropriate interventions to adjust CaF to an appropriate level. Additionally, it is desirable to develop outreach strategies aimed at preventing falls among older patients. Based on the literature reviewed, the current study posited several hypotheses. First, it was hypothesized that personality traits (especially neuroticism) may be significantly related to CaF among older patients (H1). Second, it was hypothesized that open personality traits (extraversion, openness, agreeableness, and conscientiousness) may be linked with younger subjective age (H2). Third, it was hypothesized that a younger subjective age may be associated with lower CaF (H3). Finally, it was hypothesized that subjective age partially mediates the relationship between personality traits and CaF (H4).

## 2 Materials and methods

### 2.1 Study design

This cross-sectional study was conducted at a tertiary hospital in Chengdu from March 2023 to May 2023. The aim of this study was to investigate the relationship between CaF and personality traits among older patients as well as to examine the potential mediating effect of subjective age.

### 2.2 Ethical approval

The article does not contain any clinical trials involving human participants or animals. All procedures were conducted in adherence to the applicable guidelines and regulations, including the 2013 revision of the Declaration of Helsinki. The study protocol received approval from the institutional review board prior to the commencement of the study. Before the study began, informed consent was obtained from each patients, and they were made aware of the study’s purpose, potential risks, and potential benefits. The authors assert the accuracy and comprehensiveness of the data and analyses, as well as the fidelity of the design implementation. This study was reported in accordance with the Strengthening the Reporting of Observational Studies in Epidemiology (STROBE) statement.

### 2.3 Subjects

This study comprised a convenience sample of 407 patients aged 60 years old or older. The sample size was determined based on the recommendation of 5–10 times the number of items in the Chinese Big Five Personality Questionnaire (40 items) (47) and considering a potential dropout rate of 15%, resulting in a required sample size of 235 to 471 patients. Each patient underwent routine admission assessment through an internet-based assessment system. The inclusion criteria were as follows: (1) aged 60 years or older; (2) possessed normal mobility ability; (3) exhibited normal cognitive function; (4) had complete medical records; and (5) provided informed consent and agreed to participate. To mitigate potential bias, the participation screening process was conducted by two highly skilled and experienced nurses.

### 2.4 Measures

#### 2.4.1 Personal traits

The Big Five Inventory (BFI) is a commonly utilized tool for assessing personality traits; it is known for its comprehensive coverage and concise format and has gained significant recognition since its initial publication (48). To account for cultural diversity, the Chinese Big Five Personality Inventory Brief Version (CBF-PI-B) was employed, which consists of 40 items that are hierarchically categorized into five overarching domains: extraversion, agreeableness, conscientiousness, neuroticism, and openness (49). Extraversion is characterized by high levels of energy and sociability. Agreeableness is characterized by friendly and empathic behavior. Conscientiousness is characterized by self-discipline, organization, and adherence to plans. Neuroticism is characterized by a tendency to experience frequent worry and heightened emotional instability, which manifests as more frequent and intense negative emotions. Openness is characterized by the inclination to be more accepting of novel stimuli and the ability to readily engage in a wide range of emotional experiences. Each item is assessed using a 6-point Likert scale ranging from “strongly disagree” (1) to “strongly agree” (6). For each dimension, the score ranges from 8 to 48, with a higher score indicating a greater tendency towards a particular personality trait. Scores for each trait are categorized into three levels: slight (8–21), moderate (22–35), and high (36–48). Cronbach’s alpha coefficients were calculated to assess the internal consistency of the measures for neuroticism, extraversion, conscientiousness, openness, and agreeableness. The obtained coefficients were 0.81, 0.80, 0.81, 0.78, and 0.76, respectively, indicating satisfactory levels of reliability. The subscales also demonstrated satisfactory levels of internal consistency, structural validity, and criterion correlation validity.

#### 2.4.2 Concerns about falling

The Short Falls Efficacy Scale International (Short FES-I) was derived from the Falls Efficacy Scale International (FES-I) to enhance its applicability in clinical settings and provide a more concise version for research purposes (50). The Short FES-I has been shown to exhibit excellent reliability and validity (51). It was developed as part of the Prevention of Falls Network Europe (ProFaNE) project from 2003 to 2006, which involved an intensive review of questionnaires related to the fear of falling, self-efficacy, and balance confidence (52) and aimed to measure the level of “fear of falling” or, more accurately, “concerns about falling” (CaF). It has been suggested that the Short FES-I be utilized for individuals who are at risk of falls, such as the older patients in the present study (53). The Short FES-I comprises seven items that are answered on a 4-point Likert scale. The total score is obtained by summing the scores for each item and ranges from 7 (indicating no significant CaF) to 28 (indicating severe CaF). The cut-off values for categorizing levels of CaF were as follows: 7–8 indicate low concern, 9–13 indicate moderate concern, and 14–28 indicate high concern.

#### 2.4.3 Subjective age

Subjective age was assessed using the following question: “At times, individuals may feel older or younger than their actual age. What age do you feel most of the time?.” The response indicated the individual’s “felt age.” Proportional discrepancy scores were computed by subtracting chronological age from felt age and dividing the result by chronological age (subjective age = [felt age—chronological age]/chronological age) (54). A negative score indicates a subjective age that is younger than the actual age, while a positive score indicates a subjective age that is older (equal = 0, feel younger = 1, feel older = 2).

#### 2.4.4 Confounders

Confounders were selected based on the logical sorting and screening of variables that demonstrated a significant correlation with CaF in previous studies, including demographic data, socioeconomic data, psychological parameters, chronic comorbidities, and accompanying symptoms.

Sociodemographic data included age, gender, education level (coded as primary school and below = 1, junior high school = 2, senior high school = 3, bachelor’s degree and above = 4), place of residence (coded as city = 1, countryside = 2), activities of daily living (ADLS) as measured by the Barthel Index, self-declared visual impairment (coded as no = 0, yes = 1), and self-declared falls in the last 12 months (coded as no = 0, yes = 1). Smoking status was measured by asking whether the patients smoke or not. Drinking status was measured by asking whether the patients consume alcohol (coded as no = 0, yes = 1).

Comorbidities were obtained from the medical records in the hospital information system (HIS), where doctors listed the definitive medical diagnoses. The comorbidities included diabetes, hypertension, hypoproteinaemia, anaemia, and cancer (coded as no = 0, yes = 1). The total number of comorbidities was also collected.

Accompanying symptoms were primarily self-reported and included pain, sleep disturbances, dizziness, fatigue, and loss of appetite. Patients were asked “have you felt pain/sleep disturbances/dizziness/fatigue/loss of appetite in the past month?” (coded as no = 0, yes = 1). Self-reported psychological symptoms, including anxiety, depression, and loneliness, were assessed by asking patients “have you felt anxiety/depression/loneliness recently?” Responses were provided on a scale ranging from none (0) to very much (3).

### 2.5 Data collecting

We designed electronically administered questionnaires involving required variables to collect data, through Wenjuanxing, a most commonly used online questionnaire website in China.^1^ All the interviewers were medical staff who were trained in collecting questionnaire data through face-to-face, one-on-one personal interviews. To accommodate patients who had limited literacy skills, we assigned two collectors with strong communication abilities. These collectors were responsible for explaining the research objectives, providing explanations for the items, reading the items aloud, and assisting in completing the questionnaires based on the patients’ responses. All responses were promptly reviewed and thoroughly examined to allow patients the opportunity to provide further clarification, if needed. The data were anonymized and analyzed by an independent researcher. Any patients with incomplete data were excluded.

### 2.6 Statistical analysis

Histograms and normality tests indicated that the data were not normally distributed. Therefore, categorical variables are presented as counts (n) and percentages (%), while continuous variables are reported as medians (interquartile ranges, IQRs). Multivariable linear regressions were performed to preliminarily test the independent variables impacting CaF and exam multicollinearity among the continuous variables via the Enter method. Trend tests were conducted to assess linear trends. The confounders were gradually included in the models, and thus, multiple models (both crude and adjusted weighted linear regression models) were constructed to control bias and examine the stability of the correlation. Model 0 represented the unadjusted model; Model l was adjusted for demographic data (age, sex, education level, residence, ADLs, current alcohol and smoking, visual impairment, and falls experienced within the past 12 months); Model 2 was adjusted for comorbidities that were common among older patients (diabetes, hypertension, hypoproteinaemia, anaemia, cancer, and the number of diagnoses); and Model 3 was adjusted for symptoms (pain, sleep disorders, dizziness, fatigue, inappetence, anxiety, depression, and loneliness). Finally, a mediation analysis was performed to investigate the potential mediating effect of subjective age on the association between personality traits and CaF. The 95% confidence intervals (95% CIs) were generated for all regression coefficients. Odds ratios (ORs) and 95% CIs for both direct and indirect effects were computed using the bootstrap method (55). The calculation of the mediation proportion was performed using the formula OR_DE_ (OR_IE_ − 1) / (OR_DE_OR_IE_ − 1), where OR_DE_ represents the odds ratio for the direct effect and OR_IE_ represents the odds ratio for the indirect effect. All the statistical analyses were performed using the Statistical Package for the Social Sciences (SPSS, version 20.0). All tests conducted in this study were two-sided, and *p* < 0.05 was considered to indicate statistical significance.

## 3 Results

### 3.1 Sociodemographic and clinical information

**Figure 1:**
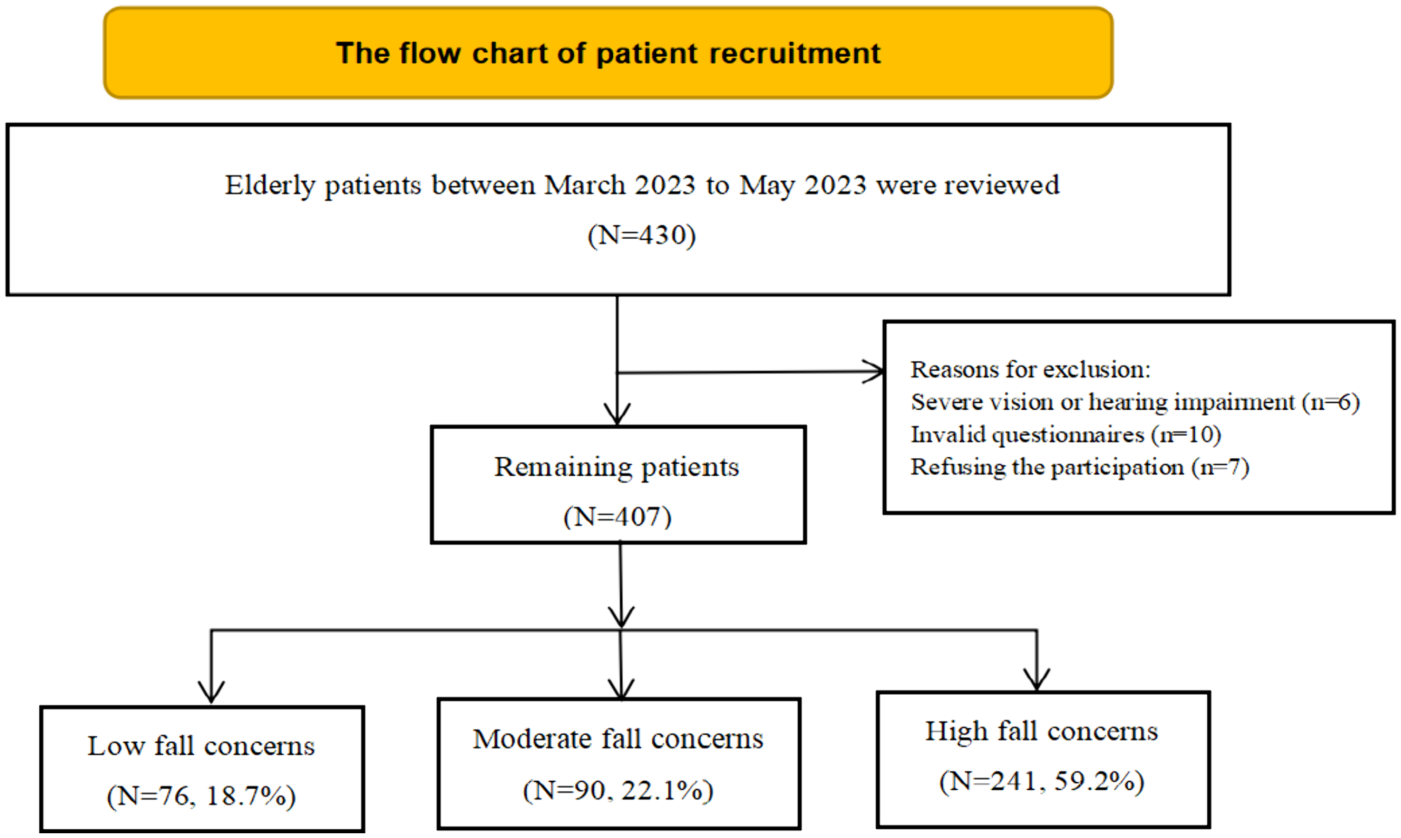
Flow chart of patient recruitment.

A total of 430 patients were included in this study. Seven patients declined to participate in the study. Six patients were excluded because of significant visual or auditory impairments. Ten patients were excluded due to having invalid questionnaires. Ultimately, 407 older individuals were included (Figure 1). Of the total sample, 76 individuals (18.7%) reported low CaF, 90 individuals (22.1%) reported moderate CaF, and 241 individuals (59.2%) reported high CaF.

The demographic characteristics of the patients are presented in Table 1. There were 214 (52.6%) males and 193 (47.4%) females. The median age was 71 (65, 77) years. The median score for the Short FES-I was 16 (12, 17). Within the past year, a total of 89 older individuals (accounting for 21.9% of the sample) reported experiencing falls. In relation to their biological age, similar proportions of patients reported feeling younger (45.2%) or older (39.1%). All of the variables were included in multiple regression models, which were conducted via the enter method. The findings revealed that neuroticism (*β* = 0.158, 95% CI: 0.048 to 0.269, *p* = 0.005), extraversion (*β* = −0.08, 95% CI: −0.15 to −0.009, *p* = 0.03), subjective age (*β* = 1.494, 95% CI: 0.99 to 1.993, *p* < 0.001), age (*β* = 0.117, 95% CI: 0.061 to 0.173, p < 0.001), education level (*β* = 0.807, 95% CI: 0.242 to 1.371, *p* = 0.005), number of diagnoses (*β* = 0.148, 95% CI: 0.01 to 0.284, *p* = 0.03), presence of pain (*β* = −1.226, 95% CI: 2.355 to −0.098, *p* = 0.03), and presence of fatigue (*β* = 1.19, 95% CI: 0.270 to 2.111, *p* = 0.01) were potentially associated with CaF among older patients.

**Table 1:** Sociodemographic data and results of multiple-variate regression.

		Concerns about falling			
	N (%)/Median (IQR)	SE	*β*	95% CI	*p* value
Personal trait					
Neuroticism	35 (32, 39)	0.056	0.158	0.048, 0.269	0.005
Conscientiousness	34 (28, 39)	0.033	0.010	−0.055, 0.076	0.76
Agreeableness	20 (14, 28)	0.032	−0.022	−0.084, 0.041	0.49
Openness	25 (20, 29)	0.041	0.058	−0.022, 0.139	0.16
Extraversion	30 (23, 34)	0.036	−0.080	−0.150, −0.009	0.03
Subjective age		0.254	1.494	0.996, 1.993	<0.001
Equal	64 (15.7%)				
Feel younger	184 (45.2%)				
Feel older	159 (39.1%)				
Female	193 (47.4%)	0.552	−0.399	−1.485, 0.687	0.47
Age	71 (65, 77)	0.028	0.117	0.061, 0.173	<0.001
Education level		0.287	0.807	0.242, 1.371	0.005
Primary school and below	155 (38.1%)				
Junior high school	146 (35.9%)				
Senior high school	82 (20.1%)				
Bachelor’s degree and above	24 (5.9%)				
Living in countryside	143 (35.1%)	0.529	0.627	−0.414, 1.667	0.24
ADLs	60 (40,85)	0.010	0.003	−0.016, 0.023	0.75
Smoking	101 (24.8%)	0.753	−0.665	−2.147, 0.816	0.38
Drinking	91 (22.4%)	0.749	0.333	−1.140, 1.806	0.66
Visual impairment	46 (11.3%)	0.679	0.617	−0.719, 1.952	0.36
History of falling	89 (21.9%)	0.511	0.173	−0.833, 1.178	0.74
Coexisting disease					
Anaemia	95 (23.3%)	0.563	0.071	−1.036, 1.178	0.900
Hypoproteinaemia	57 (14%)	0.688	−0.648	−2.002, 0.705	0.35
Diabetes	121 (29.7%)	0.490	−0.135	−1.098, 0.828	0.78
Hypertension	181 (44.5%)	0.453	0.698	−0.193, 1.589	0.12
Cancer	45 (11.1%)	0.688	0.196	−1.156, 1.548	0.78
Number of diagnoses	6 (4,8)	0.069	0.148	0.013, 0.284	0.03
Coexisting symptoms					
Pain	159 (39.1%)	0.574	−1.226	−2.355, −0.098	0.03
Sleep disorder	192 (47.2%)	0.446	−0.446	−1.323, 0.431	0.32
Dizziness	141 (34.6%)	0.488	−0.396	−1.356, 0.564	0.42
Fatigue	227 (55.8%)	0.468	1.190	0.270, 2.111	0.01
Inappetence	204 (50.1%)	0.563	0.876	−0.232, 1.984	0.12
Anxiety		0.494	0.808	−0.163, 1.779	0.10
None	224 (55%)				
Some	141 (34.6%)				
Much	36 (8.8%)				
Very much	6 (1.5)				
Depression		0.514	−0.256	−1.266, 0.754	0.24
None	271 (66.6%)				
Some	99 (24.3%)				
Much	33 (8.1%)				
Very much	4 (1%)				
Loneliness		0.395	−0.309	−1.085, 0.468	0.44
None	316 (77.6%)				
Some	71 (17.4%)				
Much	18 (4.4%)				
Very much	2 (0.5%)				

N, number of patients; IQR, interquartile range; β, regression coefficient; SE, standard error; CI, confidence interval. Continuous variables are expressed as medians (interquartile ranges), and categorical variables are expressed as n (%). Multivariate regression was conducted via the Enter method. Full details of the assignment and coding are provided as follows: subjective age (equal = 0, feel younger = 1, feel older = 2), gender (male = 1, female = 2), education level (primary school and below = 1, junior high school = 2, senior high school = 3, bachelor’s degree and above = 4), residence (city = 1, countryside = 2), smoking/drinking status (yes = 1, no = 0), visual impairment status (yes = 1, no = 0), history of falls (yes = 1, no = 0), anaemia/hypoproteinaemia/diabetes/hypertension status (yes = 1, no = 0), pain/sleep disorder/dizzy/fatigue/inappetence status (yes = 1, no = 0), and anxiety/depression/loneliness status (none = 0, some = 1, much = 2, very much = 3). Adjusted R^2^ = 0.263, F = 7.892 (P < 0.001). The Durbin-Watson statistic indicated the independence of residuals (1.785), and the histogram showed a normal distribution of residuals. There was no multicollinearity among the continuous variables (variance inflation factors were all less than 5, tolerances were all less than 1).

### 3.2 Trend tests and multivariable adjusted models

We performed tests for linear trends by entering the median value of each continuous variable in the model to assess linear trends. To control for bias and examine the stability of the correlation between variables, confounders were gradually entered into the model, and multiple models were constructed (Table 2). In the Part 1, the relationship between personality and CaF was assessed; neuroticism (*β* = 0.17, 95% CI: 0.024 to 0.31, p for trend =0.02) and extraversion (*β* = −0.07, 95% CI: −0.15 to 0.001, p for trend =0.06) were shown to be consistently correlated with CaF. In Part 2, the trend test between subjective age and CaF was conducted, and subjective age (*β* = 2.02, 95% CI: 1.28 to 2.78, p for trend<0.001) was found to be stably related to CaF. Regarding the association between personality traits and subjective age, neuroticism (*β* = 0.002, 95% CI: 0.001 to 0.004, p for trend = 0.006), conscientiousness (*β* = −0.002, 95% CI: −0.004 to 0.00, p for trend = 0.04), openness (*β* = −0.003, 95% CI: −0.01 to −0.001, p for trend = 0.003), and extraversion (*β* = −0.002, 95% CI: −0.004 to −0.00, p for trend = 0.01) were shown to be consistently associated with subjective age.

**Table 2:** The results of the trend tests and multivariable adjusted models.

		Median	Model 0 β (95% CI)	Model 1 β (95% CI)	Model 2 β (95% CI)	Model 3 β (95% CI)
Part1 Trend test regarding the relationship between personality traits and CaF						
Neuroticism	Low (ref)	16				
	Moderate	34	1.95 (−6.83, 10.72)	0.05 (−8.65, 8.75)	−0.52 (−9.19, 8.15)	−1.6 (−10.14, 6.94)
	High	40	2.7 (−6.07, 11.48)	1.03 (−7.69, 9.74)	0.51 (−8.17, 9.19)	−0.44 (−8.99, 8.11)
	P for trend β (95% CI)		0.08 0.12 (−0.01, 0.26)	0.04 0.15 (0.006, 0.29)	0.03 0.152 (0.011, 0.293)	0.02 0.17 (0.024, 0.31)
Conscientiousness	Low (ref)	20				
	Moderate	30	−0.58 (−2.23, 1.07)	−0.34 (−1.98, 1.30)	−0.37 (−2.01, 1.26)	−0.73 (−2.36, 0.90)
	High	39	0.35 (−1.32, 2.03)	0.25 (−1.44, 1.93)	0.22 (−1.46, 1.90)	−0.16 (−1.84, 1.51)
	P for trend β (95% CI)		0.17 0.05 (−0.02, 0.13)	0.40 0.03 (−0.04, 0.11)	0.40 0.03 (−0.04, 0.11)	0.61 0.02 (−0.06, 0.10)
Agreeableness	Low (ref)	15				
	Moderate	29	0.32 (−0.58, 1.22)	0.3 (−0.61, 1.21)	0.44 (−0.47, 1.36)	−0.001 (−1.0, 0.99)
	High	37	0.29 (−2.23, 2.81)	0.55 (−1.98, 3.08)	0.54 (−2.01, 3.08)	0.65 (−3.33, 2.03)
	P for trend β (95% CI)		0.48 0.02 (−0.04, 0.08)	0.47 0.022 (−0.04, 0.08)	0.33 0.03 (−0.03, 0.09)	0.88 −0.005 (−0.07, 0.06)
Openness	Low (ref)	18				
	Moderate	27	0.04 (−0.94, 1.01)	−0.04 (−0.96, 1.04)	−0.01 (−0.01, 0.99)	0.26 (−0.76, 1.29)
	High	37	−1.22 (−3.26, 0.82)	−1.16 (−3.23, 0.91)	−1.23 (−3.30, 0.84)	−1.23 (−3.29, 0.83)
	P for trend β (95% CI)		0.50 −0.03 (−0.12, 0.06)	0.53 −0.03 (−0.12, 0.06)	0.47 −0.03 (−0.13, 0.06)	0.65 −0.02 (−0.11, 0.07)
Extraversion	Low (ref)	18				
	Moderate	30	−0.69 (−1.86, 0.48)	−0.59 (−1.75, 0.57)	−0.69 (−1.87, 0.49)	−0.73 (−1.92, 0.45)
	High	38	−1.62 (−3.1, −0.14)	−1.41 (−2.89, 0.07)	−1.43 (−2.93, 0.07)	−1.52 (−3.03, −0.01)
	P for trend β (95% CI)		0.04 −0.08 (−0.15, −0.004)	0.07 −0.07 (−0.14, 0.01)	0.07 −0.07 (−0.14, 0.005)	0.05 −0.07 (−0.15, 0.001)
Part 2 Trend test regarding the relationship between subjective age and CaF						
Subjective age	Younger(ref)	−1.1				
	Equivalent	0.0	1.79 (0.56, 3.01)	1.27 (0.05, 2.48)	1.14 (−0.08, 2.35)	1.08 (−0.14, 2.30)
	Older	0.08	0.94 (2.03, 3.85)	3.13 (2.22, 4.05)	3.08 (2.16, 3.40)	2.91 (1.94, 3.88)
	P for trend B (95% CI)		<0.001 2.28 (1.55, 3.01)	<0.001 2.27 (1.55, 3.00)	<0.001 2.21 (1.48, 2.94)	<0.001 2.02 (1.28, 2.78)
Part 3 Trend test regarding the relationship between personality and subjective age						
Neuroticism	Low (ref)	16				
	Moderate	34	0.05 (0.03, 0.07)	0.04 (0.02, 0.06)	0.04 (0.02, 0.06)	0.03(0.004, 0.05)
	High	40	0.12 (0.06, 0.17)	0.11 (0.05, 0.17)	0.11 (0.05, 1.67)	0.07 (0.012, 0.13)
	P for trend β (95% CI)		<0.001 0.004 (0.002, 0.005)	<0.001 0.003 (0.002, 0.01)	<0.001 0.003 (0.003, 0.005)	0.006 0.002 (0.001, 0.004)
Conscientiousness	Low (ref)	20				
	Moderate	30	0.013 (−0.03, 0.05)	0.012 (−0.027, 0.05)	0.009 (−0.03, 0.05)	−0.01 (−0.04, 0.03)
	High	39	−0.07 (−0.05, 0.03)	−0.01 (−0.05, 0.03)	−0.01 (−0.05, 0.03)	−0.03 (−0.07, 0.01)
	P for trend β (95% CI)		0.22 −0.001 (−0.003, 0.001)	0.22 −0.001 (−0.003, 0.01)	0.20 −0.001(−0.003,0.01)	0.04 −0.002 (−0.004, 0.00)
Agreeableness	Low (ref)	15				
	Moderate	29	0.12 (−0.09, 0.33)	0.13 (−0.08, 0.34)	0.11 (−0.1, 0.32)	0.06 (−0.14, 0.26)
	High	37	0.11 (−0.11, 0.32)	0.12 (−0.09, 0.33)	0.10 (−0.11, 0.31)	0.06 (−0.14, 0.25)
	P for trend β (95% CI)		0.30 −0.002 (−0.005, 0.002)	0.88 0.00 (−0.004, 0.003)	0.83 0.001 (−0.004, 0.01)	0.87 0.00 (−0.004, 0.003)
Openness	Low (ref)	18				
	Moderate	27	−0.05 (−0.073, −0.027)	−0.05 (−0.07, −0.03)	−0.05 (−0.07, −0.03)	−0.04 (−0.06, −0.01)
	High	37	−0.08 (−0.13, −0.03)	−0.07 (−0.12, −0.02)	−0.07 (−0.12, −0.02)	−0.05 (−0.1, −0.01)
	P for trend β (95% CI)		<0.001 −0.005 (−0.007, −0.003)	<0.001 −0.01 (−0.01, −0.002)	<0.001 −0.004 (−0.01, 0.01)	0.003 −0.003 (−0.01, −0.001)
Extraversion	Low (ref)	18				
	Moderate	30	−0.044 (−0.07, −0.02)	−0.04 (−0.07, −0.02)	−0.042 (−0.07, −0.01)	−0.03 (−0.06, −0.002)
	High	38	−1.62 (−0.1, −0.03)	−0.06 (−0.1, −0.03)	−0.06 (−0.09, −0.02)	−0.04 (−0.08, −0.01)
	P for trend β (95% CI)		<0.001 −0.003 (−0.005, −0.002)	<0.001 −0.003 (−0.01, 0.00)	0.001 −0.003 (−0.01, 0.00)	0.01 −0.002 (−0.004, −0.00)

CaF: concerns about falling; ref: reference group; Model 0 represents the unadjusted model; Model l was adjusted for demographic data (age, gender, education level, residence, ADLS, current alcohol and smoking, visual impairment, and falls experienced within the past 12 months); Model 2 was adjusted for comorbidities that were common among older patients (diabetes, hypertension, hypoproteinaemia, anaemia, cancer, and the number of diagnoses); Model 3 was adjusted for symptoms (pain, sleep disorders, dizziness, fatigue, inappetence, anxiety, depression, and loneliness). Test for trend based on variables containing median values for each quintile.

### 3.3 Mediating effect of subjective age

The findings from the mediation effect analysis (Table 3) demonstrate the impact of personality and subjective age on CaF. The 95% bootstrap CI refers to the confidence interval (CI) obtained from bootstrap sampling, which represents the 95% level of confidence. A significant effect is indicated when the CI does not include zero. The results revealed that the associations of openness and agreeableness with CaF were fully mediated by subjective age. Moreover, the relationship between extraversion and CaF was partially mediated by subjective age. Subjective age did not mediate the relationships of conscientiousness or neuroticism with CaF.

**Table 3:** The results of the mediating effect of subjective age.

Models	c (95% CI)	a (95% CI)	b (95% CI)	a*b (95% Boot CI)	c’ (95% CI)	Mediation effect
N= > SA = > CaF	0.147 (0.031, 0.262) *	−0.008 (−0.030, 0.014)	1.494 (0.997, 1.991) **	−0.012 (−0.044, 0.020)	0.158 (0.048, 0.269)*	NS
C= > SA = > CaF	−0.002 (−0.070, 0.066)	−0.008 (−0.021, 0.005)	1.494 (0.997, 1.991)**	−0.012 (−0.054, 0.014)	0.010 (−0.055, 0.076)	NS
A= > SA = > CaF	−0.002 (−0.067, 0.063)	0.013 (0.001, 0.026) *	1.494 (0.997, 1.991)**	0.020 (0.003, 0.078)	−0.022 (−0.084, 0.040)	Complete effect
O = > SA = > CaF	0.027 (−0.056, 0.111)	−0.021 (−0.037, −0.005)*	1.494 (0.997, 1.991)**	−0.031 (−0.085, −0.008)	0.058 (−0.022, 0.139)	Complete effect
E = > SA = > CaF	−0.104 (−0.177, −0.031)*	−0.016 (−0.030, −0.002)*	1.494 (0.997, 1.991)**	−0.024 (−0.080, −0.000)	−0.080 (0.150, −0.010)*	Partial effect (23.2%)

^*^p < 0.05; ^**^p < 0.01; N, neuroticism; C, conscientiousness; A, agreeableness; O, openness; E, extraversion; SA, subjective age; CaF, concerns about falling; CI, confidence interval; NS, no significance; c, total effect of personality on CaF when there is no mediation variable in the model; a, partial effect of personality on subjective age; b, partial effect of subjective age on CaF; a*b, mediating effect; c’, direct effect of personality on CaF when there is a mediating variable in the model.

## 4 Discussion

Falls are often nominated as a significant concern among older adults due to their high frequency and negative impact, and they could result in repeated emergency department admissions and unplanned hospitalizations (56, 57). In the United States, in 2018, 27.5% of older adults reported experiencing at least one fall in the past year, while 10.2% reported sustaining an injury from a fall during the same period (58). In China, the incidence of falls increased substantially in older adults between 1990 and 2019 as the population aged (59). The risk of falls and the presence of CaF were found to be correlated (60). Older adults with a history of accidental falls may subsequently develop CaF (61). Unfortunately, in the present study, approximately 21.9% of patients had a fall within the past year, and no significant difference in CaF was detected between those who had a fall and those who did not have a fall in the past year (*p* = 0.74). Nonetheless, a history of falls still has been found to be a strong predictor of the incidence of CaF (61). Future studies with large sample sizes are needed. Falling has been shown to cause CaF, and CaF can also cause falls. Specifically, CaF can increase the risk of falls through the restriction of movement, changes in gait, and reduced confidence in one’s activity (62). In addition, CaF may affect a person’s fall risk through the interplay between anxiety and attention (63). Anxiety may interfere with tasks that require attention and complex coordination. High levels of CaF could require more attentional processing during multiple tasks, which detrimentally affect the efficiency of reactive stepping performance and increased the likelihood of falls (64). The current sample exhibited that approximately 22.1% expressing moderate levels of CaF and 59.2% reporting high levels of CaF. These proportions were alarming, indicating an urgent need for effective interventions to alleviate CaF among older patients. Educational strategies and motivational interventions were recommended to alter the patients’ CaF and reduce falls in the hospital (2, 7). In addition, exposure therapy (65), balance training (66), and virtual reality-based therapy (67) also exhibited satisfactory effects in terms of reducing CaF. However, there is still a lack of experimental studies targeting older patients.

Our first hypothesis (H1) was that there would be a significant correlation between personality traits (especially neuroticism) and CaF. Our study revealed a stably significant association between neuroticism and extraversion and CaF, even after controlling for confounding factors. Moreover, mediation analysis revealed that neuroticism had a direct effect on CaF, while subjective age partially mediated the relationship between extraversion and CaF. The hypothesis (H4) was partial supported. This discovery has opened up possibilities for future interventions aimed at focusing on personality and subjective age to adjust the inaccurate CaF among older patients.

Higher levels of neuroticism were positively associated with increased CaF, which could be manifested through physical, psychological, and health-related pathways. Higher levels of neuroticism were shown to be associated with more functional limitations and self-imposed restriction of activities (68), thereby contributing to frailty (69), and impaired muscle strength and consequently increasing the risk of fall among older patients. Furthermore, a decreased level of physical activity enhanced the association between higher neuroticism and poorer cognitive outcomes (70), thereby reinforcing the vicious cycle. In terms of psychological aspects, neuroticism has been found to have a strong correlation with negative emotions (71). Individuals with neuroticism tend to experience more intense and frequent negative affect (72), including distress and anxiety symptoms (38, 55). These negative emotions can lead to a sense of losing control over one’s life and lower levels of life satisfaction. Notably, anxiety is a significant psychological factor that can contribute to heightened psychological tensions, ultimately leading to high CaF in patients (73). Additionally, neuroticism has been found to increase the likelihood of developing various chronic diseases, including stroke (74), cancer (75), mental disorders, irritable bowel syndrome (76), and cardiovascular disease (77). As a result, these chronic illnesses are likely to intensify CaF and the risk of falling (4, 78). Furthermore, neuroticism has been found to lead to various adverse symptoms, including fatigue (79), pain (80), and insomnia (81), which can impair individuals’ ability to cope and their self-efficacy in daily activities and concerns (82). These findings could explain the relationship between neuroticism and older subjective age. Although subjective age was related with both neuroticism and CaF, subjective age was not found to mediate the association between neuroticism and CaF in this study. Additional analyses should be conducted in future research endeavors.

Moreover, compared with high CaF, low CaF still warrants attention because it may lead to unintentional falls due to ignorance of risk. Extraversion was negatively correlated with CaF, suggesting that patients who display higher levels of extraversion (characterized by being energetic and sociable) may be less apprehensive about falling and therefore more inclined to underestimate their risk of falling. Individuals with extraverted traits tend to experience better physical well-being in various aspects, including reduced fatigue (83), increased grip strength (84), and fewer limitations in ADLs (68). Consequently, they may have a heightened sense of independent activity and an overly confident perception of their condition, despite being at a high risk of falling. In addition, extraversion was found to be a significant predictor of increased occurrence of positive events (85), increased levels of well-being (86), and increased life satisfaction (87). These positive feelings about oneself contribute to decreased vigilance towards potential hazards or risks in daily life. Another underlying pathway was through subjective age. The mediation analysis performed herein revealed that subjective age accounted for approximately 23.2% of the total effect of extraversion on CaF. Consistent with recent literature (88), trend test analysis has demonstrated a positive association between the extraversion trait and subjective age among older individuals. Additionally, a robust significant positive correlation was observed between subjective age and CaF. This suggests that individuals who perceive themselves as younger tend to have lower CaF, thus providing support for hypothesis (H3). Younger subjective age represents a distinct manifestation of self-enhancement, higher levels of autonomy and self-efficacy. Thus, extraverted older patients are more likely to underrate and neglect the risk of falls via younger self-perception. Our study revealed that individuals with a higher chronological age expressed greater *Ca.* This could be explained by the decline in self-efficacy and self-confidence in behavioral activities due to inferior balance ability, long-term treatment, and deteriorating health conditions associated with the ageing process (89).

Considering the aforementioned information, the analysis revealed a significant relationship between extraversion, neuroticism, and CaF. Additionally, it was observed that subjective age played a crucial mediating role in the relationship between extraversion and CaF. Additionally, our research offered empirical support to substantiate the correlation between subjective age and CaF, as well as the correlation of neuroticism, conscientiousness, openness, and extraversion with subjective age. This provided new prospective evidence that personality and subjective age could help assess CaF and identify the risk of falls in older patients. The primary strength of the current study lies in the inclusion of patients who were old patients regarded as being at high risk of falls according to guidelines. Previous research has predominantly concentrated on older adults residing in community settings. Future research should initiate appropriate intervention strategies targeting hospital settings and older patients to effectively reduce hospital falls. In addition, this study considered and integrated various potential influencing factors, including sociodemographic data and health-related variables. Multiple models and trend tests were employed to mitigate bias and investigate the true correlation. Several limitations should be considered when interpreting the results of the current study. To establish primacy, the cross-sectional design does not allow for causal inferences, as the relationship may be bidirectional. Further research should employ experimental or longitudinal designs to obtain robust empirical evidence supporting the causal assumptions posited in the present study. Additionally, several self-reported parameters, such as symptoms and psychological variables (depression, anxiety, and feelings of loneliness), were analysed herein. Nevertheless, our study employed a unidimensional measure in which patients were asked a direct question regarding their subjective experience. As a result, reporting and recall bias may have affected the findings.

## Data Availability

The original contributions presented in the study are included in the article/supplementary material, further inquiries can be directed to the corresponding author.

## Footnotes

^1^ https://www.wjx.cn/

## Author’s note

We did not use AI tools to analyze nor draw insights from the data as part of the research process.
